# In Full Force. Mechanotransduction and Morphogenesis during Homeostasis and Tissue Regeneration

**DOI:** 10.3390/jcdd7040040

**Published:** 2020-10-01

**Authors:** Vasiliki Tsata, Dimitris Beis

**Affiliations:** Developmental Biology, Clinical, Experimental Surgery and Translational Research Center, Biomedical Research Foundation Academy of Athens, 11527 Athens, Greece

**Keywords:** mechanotransduction, development, regeneration, tissue-engineering, biomechanics, cardiac valves

## Abstract

The interactions of form and function have been the focus of numerous studies in the context of development and more recently regeneration. Our understanding on how cells, tissues and organs sense and interpret external cues, such as mechanical forces, is becoming deeper as novel techniques in imaging are applied and the relevant signaling pathways emerge. These cellular responses can be found from bacteria to all multicellular organisms such as plants and animals. In this review, we focus on hemodynamic flow and endothelial shear stress during cardiovascular development and regeneration, where the interactions of morphogenesis and proper function are more prominent. In addition, we address the recent literature on the role of extracellular matrix and fibrotic response during tissue repair and regeneration. Finally, we refer to examples where the integration of multi-disciplinary approaches to understand the biomechanics of cellular responses could be utilized in novel medical applications.

## 1. Mechanical Stimuli to Guide Biological Processes

During the late 1800s, scientists primarily working on bone and/or orthopedics put forward the notion that an extracellular stimulus, such as mechanical load, can instruct a specific tissue response, such as trabecular bone adaptation and remodeling [1,2,3]. We currently know that the series of events in which a physical force is converted into a cellular response, able to induce in turn a biological process, are regulated and described by the conserved principles of cellular mechanics and mechanotransduction. From bacteria that sense osmotic pressure changes through their membranes and adjust their cytoplasm [4], to plant stems that adapt their growth patterns in response to wind and rain, touching of passing animals [5] or gravity [6], to fracture healing after bone injury [7], all living organisms use stimuli originating from their environment to constantly adjust their behavior, respond to their surroundings and ensure survival.

**Figure 1:**
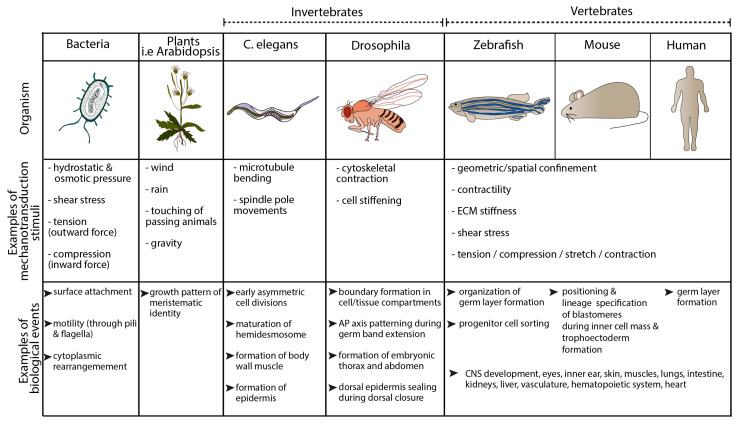
Examples of key mechanotransduction stimuli and occurring cellular events from bacteria to humans.

With forces applied to a given cell from seconds to hours, ranging from pico- to nano- newtons and external stimuli spanning changes in i) the stiffness of extracellular matrix (ECM), ii) air and osmotic pressure, iii) compression, contraction, and stretch, iv) shear stress and v) fluid flow, cells respond to stimuli by adapting their cell function and, therefore, key cellular processes such as gene transcription, protein synthesis, proliferation, migration, differentiation and/or apoptosis. This renders mechanotransduction crucial to development, homeostasis, disease progression and regeneration (Figure 1).

In this review, we provide an overview of the basic principles that allow cells to sense and integrate mechanical cues and discuss how mechanotransduction shapes morphogenetic events during animal and tissue development with a specific focus on cardiogenesis. Furthermore, we summarize how different types of biomechanical signaling orchestrate tissue remodeling during homeostasis and regeneration.

## 2. Sensing and Integrating Mechanical Forces

Mechanotransduction is a complex, multi-step reaction cascade. The first steps involve mechanotransmission and mechanosensing: the transmission of an external, mechanical cue to a mechanosensitive cell component and its subsequent local, active perception by the sensor cell. During the next step, described as mechanocoupling, stimuli surpassing a certain threshold are transduced into an intracellular change; this might be a biochemical signal and/or electrochemical activity and is, therefore, referred to as biochemical coupling. The activation of such a signaling cascade induces the mechanoresponse of the sensor cell, which is eventually transferred to an effector cell and initiates its response.

So how does a mechanical stimulus transform into a subcellular, molecular signal with such high efficiency? Cells sense, control and interpret external cues, whether they come from the environment or a neighboring cell, by integrating changes in surface parameters through their membranes. Due to its lipid bilayer composition, besides its role as a physical barrier that selectively allows the movement of ions and molecules in and out of cells, the cell membrane provides an excellent dock for a variety of mechanosensitive molecules. A plethora of different biological structures, such as ion or cell–cell junctional channels and transmembrane receptors, have been suggested as mechanosensors and can be found in many different cell types. The integration of external cues originating from the ECM occurs through highly specific cell–matrix interactions. These take place in specialized sites, where cell membranes harbor the ECM in structures of specific molecular composition [8], named focal adhesions or focal contacts [9]. In turn, these form multi-protein complexes [10] that connect the extracellular space to the cell’s interior and the cytoskeleton, through transmembrane receptors such as those of the integrin family [11,12]. The extracellular part of the integrin receptor anchors defined ECM proteins, like various proteoglycans [13,14], while the cytoplasmic tail interacts with proteins of the cytoskeletal network, such as actin, myosin, tubulin, and paxillin [15]. The latter can further act as a scaffold and recruit other structural and signaling elements like vinculin [16,17] and/or Focal Adhesion Kinase (FAK) [18,19], one of many protein tyrosine kinases (PTKs), respectively. This creates a highly dynamic and tightly regulated complex that effectively creates a physical continuity between the extracellular space and the intracellular environment.

**Figure 2:**
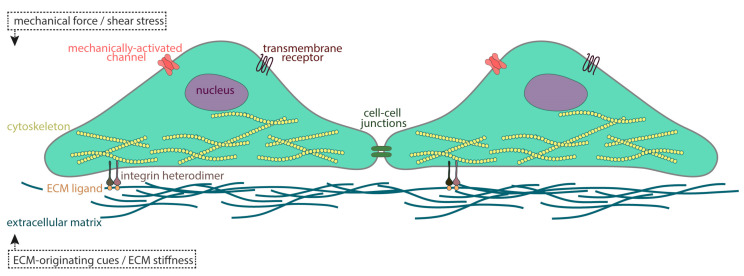
Integration of external stimuli. The perception and interpretation of external signals starts at the cell membrane. Cells sense changes in their surface parameters through transmembrane receptors, ion or cell–cell junctional channels. Sensing of external cues originating from the extracellular matrix (ECM), takes place in specialized sites of interaction between the ECM and the membrane.

Conformational changes are the predominant mechanism through which these protein interactions can activate intracellular signaling cascades, mediating in turn the transmission of mechanical information into biological responses. For example, spatial rearrangements affect the folding landscape of macromolecules, the accessibility of their molecular recognition sites [20] and, therefore, their function. Different signaling pathways have been recognized as early effectors of mechanotransduction, with tyrosine kinase activities and small GTPases playing a crucial role in the regulation of the matrix–integrin–cytoskeleton complexes [21,22]. Once a tyrosine kinase, like FAK, is recruited to the focal adhesion complex, phosphorylation on its tyrosine residues modulates its catalytic activity [23]. This directs the creation of a high-affinity binding site for additional PTKs, such as those of the Src-family of protein tyrosine kinases (SFKs) and, thereby, of a kinase complex that can in turn regulate the activation of downstream signal transduction pathways, such as Ras [24,25,26]. Proteins of the Ras signaling pathway, also known as the Ras/Raf/Mitogen-activated protein kinase/ERK kinase (MEK)/extracellular-signal-regulated kinase (ERK), can act as the connecting elements between cellular compartments [27]. The solidity of this macromolecule complex is further strengthened by membrane protein-adaptors, such as caveolin-1, that connect integrins to the tyrosine kinases and provide a scaffold that ensures the efficient recruitment and separation of the appropriate signaling molecules [28,29]. Hence, mutations on tyrosine residues greatly affect the activation and subsequent stability of the complex, and, therefore, the eventual translocation of the initial stimulus to the nucleus (Figure 2).

### 2.1. From the Cell Surface to the Core

Signal propagation depends on the transport of the initial stimulus from the cell membrane to the DNA, in a chain-like reaction, such that information originating from cell surface-tethered, transmembrane receptors will be coupled to transcription factors that regulate and potentially alter gene expression. As discussed in the previous section, the transmission of information involves the propagation of biochemical signals through intracellular signaling components and secondary messengers. As signals need to be coupled to gene expression changes, the endpoint of the signaling cascade is the nucleus, where the translocation of certain co-factors activated in the pathway will activate the appropriate transcription factors. Alternatively, biochemical signaling can occur with the direct movement of transcription factors from the cell surface or cytoplasm to the nucleus. This is the case for the mechanosensitive proteins paxillin, β-catenin, and Notch and the cytoplasm-sequestered transcription nuclear factor kappa-light-chain-enhancer of activated B cells (NFkB), signal transducer and activator of transcription 3 (STAT3) and nuclear factor of activated T-cells (NFAT). Another option, would be the activation of a transcription factor shuttling host. Some of the most described mediators are the nuclear transducers Yes-associated Protein (YAP) and transcriptional coactivator with PDZ-binding motif (TAZ) [30]. YAP/TAZ dynamically respond to increasing applied forces by rapidly adjusting their nuclear to cytoplasmic localization [31]. As signals need to travel from the margins to the inner core of the cell either by diffusion or active transport, transmission of biochemical signals can range from seconds to minutes or even hours.

**Figure 3:**
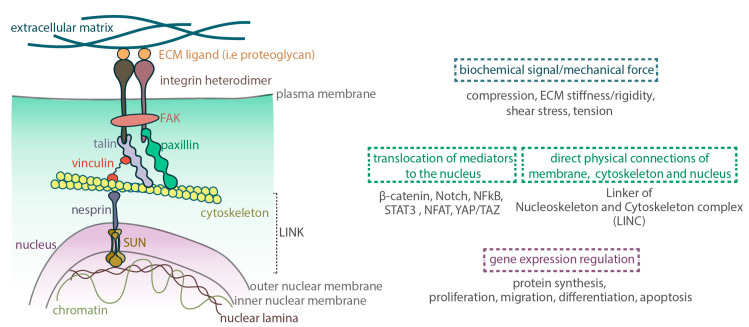
Signal propagation to the nucleus. Biochemical and mechanical stimuli are transferred to the nucleus and become effective through i) direct physical interactions of the membrane, the cytoskeleton and the nucleus and/or ii) the translocation of activated mediators from the cytoplasm to the nucleus. Eventually, stimuli surpassing a certain threshold can instruct transcriptional changes and regulate the gene expression pattern of a given cell.

In the case of minimal physical forces, signal transduction can occur through the direct physical connections between the membrane, the cytoskeleton and the nucleus. In contrast, signals bearing a great amount of mechanical tension, require the remodeling of the cytoskeleton and the reorganization of perinuclear actin network, thus, allowing changes to nuclear shape and morphology. The magnitude of the force and the extent of cytoskeletal reorganization needed until the signal reaches the nucleus, defines the timescale of propagation which, in this mode, can reach sub-millisecond levels. The mechanical coupling and physical association of the cytoskeleton to the nucleus occurs through the Linker of Nucleoskeleton and Cytoskeleton (LINC) complex [32]. This comprises a protein network that not only ensures nuclear positioning and provides stability but also links the nuclear envelope to the cytoskeleton and the nuclear lamina [33]. Coupling connections occur through nesprins, proteins of the outer nuclear membrane [34,35] that bridge through their interactions the extracellular space to the nucleus and, subsequently, the genome. Towards the inner side of the nucleus, nesprins bind to SUN proteins, that are located in the inner nuclear membrane and interact with nuclear intermediate filaments, lamins [36,37]; towards the outer side, nesprins associate with actin and microtubule filaments [38]. As lamins anchor chromatin domains and actively take part in the assembly and organization of DNA machinery [39,40], this highly organized complex has the ability to instruct changes in the gene expression pattern of a given cell (Figure 3).

### 2.2. Here Comes the Nucleus

Mechanical stimuli reach the nucleus and become effective by means of transcription factors. These bind to regulatory DNA regions, enhancers or promoter sites, and control the up- or down- regulation of given genes. The spatial organization of chromosomes plays a crucial role in how such signals can guide transcriptional regulation and orchestrate nuclear mechanotransduction [41]. The density and compaction of the double-stranded DNA, represent a major obstacle that limits the accessibility of nuclear elements to such interactions. Recent accumulating evidence has shown that this is an active process. The nucleus, a mechanosensitive organelle itself, does not only respond to the downstream effectors of mechanical cues, but also to applied forces directly [42,43,44].

So how do transcription factors find their way through the highly packed chromatin landscape of the nucleus? The 3D organization of chromosomes plays a crucial role in the regulation of gene expression. To circumvent accessibility restraints placed on transcription factors by the highly dense, super-coiled DNA, it has been shown that chromosomal territories, representing sites of active transcription of co-regulated, functionally-related genes, are organized in a spatially defined, neighboring manner [45,46]. This chromosome intermingling allows the identification of distinct “mechanical hotspots for transcription” [47] and is strongly correlated with the common and efficient transcriptional control of functionally-clustered genes [48,49,50], as well as the maintenance of transcriptional memory to dividing cells.

## 3. Mechanical Forces Regulating Embryo and Tissue Development

The conversion of mechanical signals into gene expression regulation directs a plethora of fundamental biological processes that shape both embryonic development and adult tissue homeostasis. From the maturation of the ovary and throughout gametogenesis [51,52] to egg activation [53,54,55] and early asymmetric cell divisions, forces are generated and guide developmental patterning and tissue morphogenesis across species. In *Arabidopsis*, the expression of the homeobox gene *SHOOT MERISTEMLESS (STM*), key determinant of meristematic identity, is correlated to the curvature of the shoot apical meristem and induced by mechanical stress applied on the boundary domain [56], while the fertilization of the horseshoe crab (*Limulus Polyphemus)* egg requires the activation of the egg upon a spring force which is stored and applied by the sperm acrosome. The extension of an actin-based bundle [57], allows the sperm to penetrate the vitelline layer and triggers the cytoplasmic release of Ca^2+^ that powers fertilization [58]. Similarly, combined forces that occur by movements of the spindle pole and microtubule bending, contribute to the early asymmetric cell divisions shaping the *Caenorhabditis elegans* embryo [59], while maturation of the hemidesmosome structure into a junction that will later on direct the formation of body-wall muscle and the epidermis, occurs via the activation of a mechanotransduction pathway operating on a Rac GTPase and the phosphorylation of intermediate filaments [60]. In *Drosophila*, the stiffening of cells due to forces generated by myosin II contraction and exerted between neighbor cell relationships, i) sets the boundaries between different cellular/tissue compartments [61], ii) directs the patterning of anterior–posterior axis during germ band extension and formation of the embryonic thorax and abdomen [62,63], and iii) drives the sealing of the dorsal epidermis during dorsal closure [64]. Moreover, during *Xenopus* gastrulation, involution of the mesoderm requires the tension-dependent assembly of a fibronectin-based extracellular matrix. This guides the collective migration of cells towards the posterior side during convergence, a series of cellular movements that will eventually narrow and extend the embryo along the mediolateral body axis. Intriguingly, the migration of cells depends on their cadherin-mediated cell–cell contacts and can only take place when the mesoderm and the notochord remain stiff to prevent tissue collapsing or deformation [65,66]. Along the same lines, the mechanosensitive phosphorylation of β-catenin–tyrosine-667 and its subsequent nuclear translocation at the onset of gastrulation are essential for the expression of *notail*, a mesoderm-inducing gene in the zebrafish embryo [67], while regulation of actomyosin-dependent cell-cortex tension by Nodal and transforming growth factor β (TGFβ)-signaling drives progenitor-cell sorting and the organization of germ-layer formation [68]. Similarly, in the developing mouse embryo, the contractility-dependent localization of Yap drives positioning and lineage specification of blastomeres during formation of the inner cell mass (ICM) and the trophoectoderm [69], very much in the same way as human embryonic stem cells (hESCs) self-organize to generate all three germ layers under geometric confinement and in response to bone morphogenetic protein 4 (BMP4) [70].

Taken together, the paradigms discussed above highlight how physical and mechanical stimuli orchestrate embryogenesis and tissue patterning across species. Intriguingly, they illustrate how the continuity between cellular components like the membrane, the cytoskeleton and the nuclear envelope, allows the transduction of the stimuli to the effector endpoint, the DNA, and the transformation of the initial force into gene expression regulation. In fact, the ability of cells to sense and respond to mechanical forces, while controlling their mechanical properties, is highly dependent on this exact cellular architecture, described first in architectural and now also biological terms, by tensegrity (tensional integrity) [71,72,73]. This model views the complex cellular cytoskeletal network as an architectural structure in which stability is ensured by the tensile forces that its interacting components: the ECM, the focal adhesions, the filaments and the microtubules generate and oppose each other to achieve cellular balance and stability. This tensional prestress in which cells are found, allows them to not only control their shape and structure but also channel incoming forces into regulation of cell and whole-tissue behavior.

### 3.1. The (Mechano)Sensitive Heart

All organs are sensitive to mechanical signals. In vertebrates, mechanotransduction occurs in all tissues such as the central nervous system (CNS) [74] and the eyes [75], the inner ear [76] and the skin [77], the muscles [78], the respiratory [79] and intestinal tract [80], the kidneys [81], the liver [82], the vasculature [83] and the hematopoietic system [84]. The heart however is a paradigm where form and function can be best studied. Particularly, in the context of how mechanical forces shape cardiac development and morphogenesis [85].

The heart is the first functional organ to develop during vertebrate embryogenesis. In humans, cardiogenesis begins very early, at around 18 to 19 days post fertilization with the initial formation of two cardiogenic regions arising from the lateral splanchnic mesoderm, one on either side of the neural plate, at the anterior end of the early embryo. As folding occurs, these two primary heart fields will transit from two oval-shaped structures to two endocardial tubes that will eventually converge towards each other and fuse together at the embryonic midline, forming the primitive heart. These initial steps of cardiac development involve conserved signaling pathways across vertebrate species. However, the timing of events differs significantly between them. In zebrafish, for example, the first cardiac contraction occurs 24 h post fertilization (hpf), while more than 8 and 24 days are required in mice and humans, respectively (reviewed in [86]). The cardiac tube, initially valve-less [87], will quickly bend, rotate and invert its spatial organization (cardiac looping) while differentiating into more complex and distinct parts. As the newly developed heart is required for meeting the circulatory oxygen and nutrient demands of all other developing embryonic tissues and organs, including the vasculature, peristaltic contraction begins during early looping events. Although it has been proposed that initiation of heart contraction is not coupled to the nutritional support of other developing tissues, rather than merely facilitating cardiac maturation and angiogenesis [88], the entity of morphogenetic events taking place throughout cardiogenesis are intermittently interwoven to hemodynamic changes. The latter have been shown to regulate cardiac cell proliferation, differentiation and growth, in close synergy to genetic factors [89].

### 3.2. Fluid Dynamics and the Role of Endocardium

As cardiac looping and remodeling evolve, the heart obtains its three-dimensional structure and becomes a curvature-based organ. Concomitantly, the vascular walls begin to accommodate an elevated degree of shear stress due to blood flow. Both the primitive heart tube as well as the mature cardiovascular system are lined by the endocardium, an inner layer of endothelial cells that comes in direct contact with the vascular wall and the blood flow. Hence, the endothelium acts as the sensor and signal transducer of the biomechanical stimuli generated by flow, including shear stress, stretch strain and hydrostatic pressure [90,91]. Interestingly, an increase in the amount of fluid shear stress placed on cultured monolayers of bovine endothelial cells has been shown to activate a K^+^ selective, shear-stress activated ionic current [92]. Moreover, this results in time-dependent marked changes in (i) cell shape and cytoskeletal assembly, (ii) basic endothelial functions such as fluid endocytosis [93] and (iii) differential regulation of endothelial-derived factors like endothelin-1 mRNA [94]; indicating that fluid mechanical forces can directly influence endothelial cell physiology. It has been shown that endothelial cells sense fluid dynamics through a mechanosensory complex involving (i) the immunoglobulin family receptor platelet endothelial cell adhesion molecule (PECAM)-1 that transmits the mechanical force through Src activation, (ii) the vascular endothelial cell cadherin (VE-cadherin) that acts as an adaptor element binding directly to the transmembrane domain of VEGFR2 [95] and (iii) the vascular endothelial growth factor receptor 2 (VEGFR2) that subsequently activates phosphatidylinositol-3-OH kinase (PI(3)K) [96]. Activation of the complex triggers the conformational activation of integrin αvβ3 which mediates its binding to ECM proteins and the cytoskeletal remodeling and alignment of endothelial cells in the direction of flow [97,98]. Interestingly, a certain threshold/set point of fluid stress is needed to activate the transduction cascade [99], indicating a fine balance between the cues that a cell receives and the output responses these will generate. Moreover, Piezo1 (Fam38a) channels have been also shown to act as integrators and sensors of frictional forces arising from fluid flow. When negative pressure was used to deliver a physical force in cell-attached membrane patches of mouse embryonic endothelial cells, unitary single channel events (with ionic characteristics representative of Piezo channels) were detected within less than 1s. Importantly, Piezo1 channel activation was coupled to elevated Ca^2+^ influx and the downstream activation of calpain-2 [100], a cysteine protease known to control the turnover of focal adhesion anchorage [101]. Piezo is also shown to regulate Kruppel-like factor (*klf*)*2* activity in the endothelium as well as Yap1 localization in the smooth muscle cells in the context of zebrafish outflow tract development, providing an example of how mechanotransduction can occur between cell types during valvulogenesis [102]. Overall, these data suggest a strong correlation between hemodynamics and endothelial cell responses and argue in favor of an imminent role for mechanical forces and blood flow pattern in shaping cardiac development and homeostasis.

So far, a great amount of in vitro studies has elegantly shown how endothelial cell behavior is regulated by mechanical cues. Yet, there is an inherent difficulty in mapping intracardial flow dynamics *in vivo*. In the last few decades, the relevance of shear forces in cardiac function in vivo*,* has been greatly advanced with the use of the zebrafish (*Danio rerio*) as a model organism. Due to their (i) external fertilization, (ii) small size, (iii) high degree of conservation with the human genome [103] and (iv) their amenability to forward and reverse genetic approaches [51,104], zebrafish have emerged as an ideal model organism to study the cellular and molecular events orchestrating cardiogenesis in vivo [105]. Furthermore, because of their small size, zebrafish embryos receive sufficient oxygen by passive diffusion which, in contrast to other model organisms, allows them to develop normally even in the total absence of active blood circulation [106]. Consequently, the cellular and molecular basis of cardiovascular defects in which blood flow is perturbed, can be studied in great detail [107]. Finally, due to their high ability to regenerate many tissues [108], including their heart [109], zebrafish are widely used to unravel the mechanisms governing tissue repair and functional recovery in a regeneration-competent vertebrate.

### 3.3. What Can Fish Teach Us about Cardiogenesis

Together with their small size, a great advantage of zebrafish embryos for the investigation of cardiac morphogenesis, is their optical clarity that allows the in vivo visualization of intracardiac dynamics in the developing beating heart, in living animals. Moreover, understanding on how hemodynamics shape cardiac development, was also greatly advanced by the development of selective plane illumination microscopy (SPIM) [110,111]. The efficient optical sectioning of internal cardiac structures deep into the tissue and the generation of multidimensional images, enabled the fast, non-invasive and long-term imaging of live animals at single-cell resolution [112,113].

Indeed, high-speed imaging and digital particle image velocimetry (DPIV), revealed an extremely elevated wall shear stress, as a result of the highly viscous flow, and identified the presence of vortices in the circulation pattern during the development of the embryonic zebrafish heart [114]. Interestingly, interference with blood flow resulted in severe cardiac phenotypes that comprised the absence of cardiac looping and bulbus formation as well as defects in the development of inflow and outflow tracts [114]. Blood flow and shear stress promote embryonic haematopoiesis [115] and epithelial–mesenchymal transition (EMT) in the context of the tumor microenvironment by downregulating ERK and glycogen synthase kinase 3 beta (GSK3β) [116]. Zebrafish mutants and manipulations of circulation in vivo during embryogenesis showed that, in the absence of intracardiac sheer-stress, atrioventricular endocardial cells remain quiescent and fail to undergo a mesenchymal transition and become interstitial, resulting in cardiac valve defects [109,117]. Similarly, blood flow was shown to regulate the formation of the developing ventricle by affecting cardiomyocyte morphology. By utilizing a zebrafish *weak atrium (wea)* mutant, in which the lack of atrial sarcomeres results in impaired atrial function and decreased blood flow, Auman et al. showed that the reduction of circulation impairs cardiomyocyte elongation and enlargement, thus, impeding the formation of ventricle curvature, a key determinant to a mature and functional heart [118]. These results were strongly corroborated by Kalogirou et al., in which a *wea* homozygous mutant could be raised and investigated to adulthood [117]. The adult heart exhibited only one functional chamber, the ventricle, which was markedly enlarged, while the pattern of transvalvular flow through the atrioventricular (AV) valve was also altered. Interestingly, the AV valve presented fewer cells compared to controls and failed to mature from two to four cuspids, indicating the strong coupling of intracardiac flow dynamics to cardiac chamber formation and also valve morphogenesis (reviewed in [119]).

Furthermore, optical mapping of the *silent heart* (*sih^b109^*) zebrafish mutant, that fails to contract due to a mutation in the *cardiac troponin T* (*tnnt2*) gene and exhibits cardiac conduction defects, identified loss of contraction and also blood flow as the possible etiology behind the downregulation of Connexin-40 (Cx-40) and hence, an impairment in trabeculae formation [120]. Interestingly, the endocardium-specific, blood flow-dependent activation of Notch1 signaling and its downstream effectors *ephrin b2a* (*efnb2a*) and *neuregulin 1* (*nrg1*) were also shown to direct trabeculation in the developing zebrafish heart [121,122]. Specifically, these data also demonstrated that detection of fluid shear stress by primary cilia is required for the response of endocardial cells to cardiac contraction.

**Figure 4:**
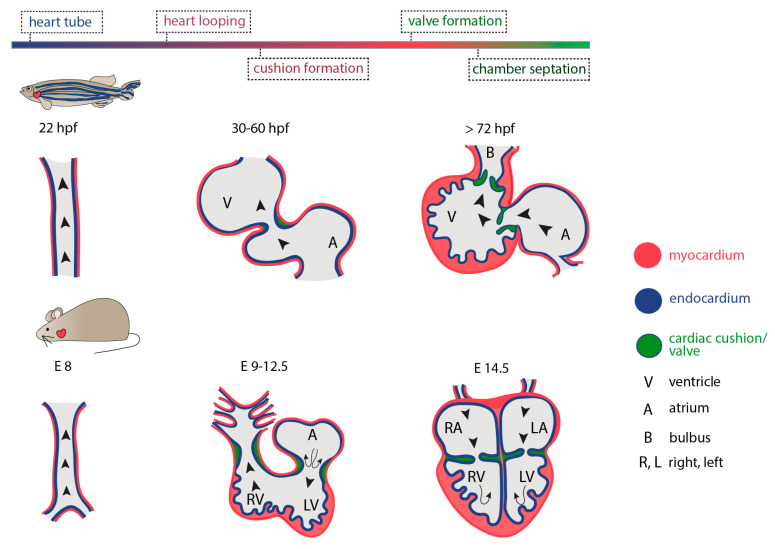
Hemodynamics and stages of cardiogenesis in zebrafish and mammals. In both zebrafish and mammals, shear forces that occur from the oscillatory/retrograde blood flow and are driven by cardiac contractility and heart looping, direct valvulogenesis. Hence, cardiac valves are formed following the initiation of heart function. Valve cells originated from endocardial cells forming cardiac cushions. Valve Endothelial Cells (VECs) and Valve Intersitial Cells (VICs) can be identified in both systems. In zebrafish, embryonic cushions give rise to cardiac leaflets that invaginate and generate cardiac valves. In contrast, in mammals, endocardial cells undergo an endothelial-to-mesenchymal transition, delaminate generate cardiac valves upon invasion into the cardiac jelly. The outer layer of epicardial cells and the cardiac jelly is omitted in all schemes for figure clarity.

A crucial step in cardiac development, is the formation of heart valves. As cardiac output is necessary to drive blood circulation both during development and adult stages, the precise formation of heart valves that prevent retrograde blood flow, plays a crucial role in a functional heart. During early cardiac development, valve leaflets originate from AV canal endocardial cells that experience high shear stress forces as a result of blood circulation and the presence of reversing-oscillatory flow patterns between the atrium and the ventricle [123,124]. To examine the relationship between hemodynamics and valve development, Vermot et al. manipulated blood flow by interfering with hematopoiesis and heart rate [125]. By altering blood flow patterns, authors identified a transcription factor from the Kruppel-like factor (Klf) family, *klf2a*, and confirmed its expression in valve precursors in response to oscillating fluid flows, thereby indicating its involvement in early events of valvulogenesis. It is now shown, that Trpv4, a mechanosensitive ion channel that is specifically expressed in the endocardium, regulates the release of endocardial Ca^2+^ and the expression of the flow-responsive *klf2a* promoter, while its absence results in prominent valve impairments [126]. This argues in favor of a prominent mechanotransduction pathway as key determinant to zebrafish valve formation. In 2013, Banjo et al. showed that the signaling cascade underlying the transmission of physical forces through *klf2a* activation during valve formation, is mediated by miRNAs. The authors pondered on previous studies demonstrating the contribution and involvement of miRNAs to fluid flow-dependent angiogenesis in zebrafish [127] and postulated that *miR-21* microRNA directs valvulogenesis in the developing zebrafish. Indeed, they showed that *mir-21* morphants do not develop heart valves. Further they demonstrated that flow-dependent expression of *mir-21* controls cell proliferation of the valve-forming endocardium via inhibition of *sprouty2 (spry2)*, *programmed cell death 4b* (*pdcd4)* and *phosphatase* and *tensin homologue B* (*ptenb)* targets, through the removal of suppression on the RTK/Ras/ERK pathway. Intriguingly, miR-21 was not essential to the stress-induced cardiomyocyte and/or fibroblast remodeling occurring in the mammalian heart [128], suggesting a potentially different mechanism through which microRNA-21 might act in different tissue and species contexts. Taken together, a long line of emerging evidence demonstrates that both morphogenesis and function of the developing and adult zebrafish heart are coupled to mechanical forces that are generated from blood circulation and can regulate the genetic basis of cell behavior within the cardiac tissue (Figure 4). But how do (cardiac) cells adjust their behavior in the absence of mechanical stimuli to maintain tissue homeostasis and remain quiescent?

## 4. Quiescence, Mechanical Stiffness and Tissue Regeneration

In zebrafish *silent heart* mutants, a basic embryonic angiogenic pattern is laid down even in the total absence of blood flow. However, endothelial cells remain quiescent and they do not form a proper lumen. Renz et al. suggested that in the zebrafish embryo, endothelial quiescence in the absence of blood flow-induced mechanical forces is safeguarded by cerebral cavernous malformation (CCM) proteins that act upstream of the Klf2 signaling pathway [129]. Using *ccm* mutants, the authors showed that CCM proteins, which are involved in cell–cell junctions and adhesion of endothelial cells to the extracellular matrix, prevent angiogenic overgrowth by negatively regulating the β1 integrin-dependent overexpression of *klf2* and the consequent upregulation of epidermal growth factor-like domain 7 gene (*egfl7)* [130]. The latter, a secreted protein that is expressed by endothelial cells and associated with the blood vessel extracellular matrix [131]. CCM proteins are crucial for the mechanosensitive properties of the endocardium during valve development [132]. Intriguingly, restoring blood flow in a zebrafish model of CCM1 has been shown to suppress its pathological phenotypes and prevent cardiovascular anomalies [133]. For a detailed review on how blood flow affects vascular development, one of the paradigms to study the interactions of form and function we encourage the readers to also consider [134]. Such mechanotransduction feedback loops are particularly relevant in the context of tissue repair and regeneration, when cells need to rapidly adjust their properties and transition from quiescence to proliferating and differentiating states to ensure survival and functional recovery of the injured tissue.

In the adult mammalian body, a variety of tissues such as the epidermis, the blood or the intestine are able to regenerate even on a daily basis, while other tissues, mainly quiescent, like the lungs or the liver can rapidly respond in cases of injury or cellular loss and replace the lost part [135]. On the contrary, the adult heart and the adult CNS, consisting of the brain and the spinal cord, have a very low regenerative capacity [136,137,138]. Interestingly, it has been shown that cells of the CNS are able to regenerate when in the periphery [139]. This suggests that a combination of post-injury events renders the mammalian CNS (and potentially other tissues) as non-permissive to regeneration. It has been shown that the experimental activation of the YAP/TAZ signaling pathway in rodents can promote regeneration in organs with limited regenerative capacity [140], suggesting a prominent role of mechanotransduction in the regeneration of different adult tissues. YAP/TAZ integrates multiple signaling pathways and is activated by the Hippo pathway as well as biomechanical forces through a yet unidentified mediator/sensor. The downstream activators and regulation in regeneration as well as cancer progression appear to be cell and tissue specific (reviewed in [141]).

Both in the adult mammalian CNS and the heart, the formation of a fibrotic scar after injury is one of the key events impeding functional regeneration, representing a chemical but also physical barrier to cell regrowth [142,143]. In zebrafish, cardiac fibroblasts are transiently activated following cardiac injury to contribute ECM components to the injury site [144]. In addition, macrophages have been reported to contribute collagen in a cell-autonomous manner at the heart injury site, both in zebrafish and mice [145]. Modulating the time-window of fibrotic response, its levels and the ECM components contributing to the scar formation might hold some answers to the regenerative potential differences between organs as well as species [146]. Moreover, similarly to cardiogenesis, local substrate stiffness critically directs axon pathfinding during CNS development [74]. Hence, certain analogies can be drawn for the two systems. Intriguingly, using atomic force microscopy in the injured rat CNS, Moeendarbary et al. have shown that, contrary to other tissues, CNS significantly softens after injury, while alteration of tissue elastical properties correlates to the expression levels of scar elements like intermediate filaments and extracellular matrix components [147]. Further, in vitro data have shown that neuron branching decreases in response to increased substrate stiffness [148] while substrate stiffness over the same range, results in higher adherent area of astrocytes, key players of the fibrous scar [149]. Alterations to substrate stiffness were also observed upon injury in the regenerating zebrafish spinal cord [150]. Using AFM-enabled nano-identation to determine elastic moduli on living spinal cord sections of control and injured samples, the authors provided the first quantitative mapping of spinal cord tissue stiffness upon injury. Intriguingly, the authors showed that in contrast to mammals, spinal cord tissue transiently stiffened during regeneration, suggesting a contradictory role of environmental mechanical cues in the regeneration-permissive environment of zebrafish. Interestingly, the mechanical differences observed in the regenerating zebrafish spinal cord correlated with changes in tissue architecture and specifically cell number densities, axonal orientation and vascularization. Taken together, these studies suggest that the finely tuned interaction between cells and their environment defines important cellular functions and argues in favor of a key role of extracellular physical properties in promoting versus inhibiting regeneration. This information could be of great importance in the identification of novel targets to be used in new therapeutic interventions.

## 5. Perspectives: Biomaterials and Tissue Engineering to Promote Tissue Regeneration

Regeneration describes the regrowth and replacement of a lost cell, tissue, organ or body part, such that the full restoration of its previous biological function is achieved. It has been an attractive field of interest for scientists already from the 1700′s when the first scientific studies on crayfish, amphibian and hydra regeneration were described [151]. A recurrent theme in tissue regeneration is the re-activation of crucial developmental signaling pathways as shown with an inducible cardiac valve injury model [152,153]. Following cardiac valve injury, the intracardiac flow pattern is reminiscent of the immature, embryonic one and results in the induction of developmental pathways such as Notch [152]. In addition, cell cycle re-entry of valve endocardial cells and ECM production following decellularization of cardiac valves is dependent on TGF-β activation, revealing a pro-regenerative role for TGF signaling [153]. Yet, to date, the basic mechanisms governing tissue regeneration are still being explored and the extent to which these can be expanded in non-regenerating environments/tissues during adulthood remains unknown. Besides the presence of a regeneration-permissive environment, successful tissue regeneration requires either the activation of endogenous stem cells or the transplantation of stem and/or progenitor cells that will replace and repair the injured tissue. However, conventional in vitro methods used so far often lack the level of complexity needed to fully recapitulate the complex ECM network which cells would physically encounter *in vivo*. The field of biomaterial science and tissue engineering is furthering our understanding on how extracellular physical properties direct stem cell homeostatic and injury responses such as quiescence, proliferation and differentiation. In silico models to predict the behavior of tissue-engineered valves has been already successfully integrated in their design [154]. In addition, the use of porous collagen-based scaffolds is shown to enhance the regeneration in experimental animal models of spinal cord injury [155]. All these advances, coming from cross-disciplinary approaches, unravel the complex cell–matrix interactions we need to understand, in order to achieve optimal regeneration. Specifically, the emergence of novel cultured conditions comprising hydrogels with tunable stress properties, 2D and/or 3D synthetic matrices, microfluidic chambers and cellular scaffolds, allows the precise and timely control of the biomechanical properties of the stem cell niche microenvironment and, hence, of potential external cues determining cell lineage fate (also reviewed in [156]**)**. Such approaches can unravel several of the necessary elements (cells, secreted factors, extracellular components) as well as the mechanical properties required for successful tissue repair in “difficult” to regenerate cellular environments.
